# Acute Calcific Tendinitis of the Longus Colli Muscle Masquerading as Acute Meningitis: A Case Report and Review of the Literature

**DOI:** 10.7759/cureus.43400

**Published:** 2023-08-13

**Authors:** Li Anne Cheong

**Affiliations:** 1 General Medicine/Internal Medicine, Sengkang General Hospital, Singapore, SGP

**Keywords:** odynophagia, meningitis, fever, acute neck pain, acute calcific tendinitis of the longus colli muscle

## Abstract

We present a case report of a young female who presented with acute neck pain, odynophagia, and fever. These symptoms were suggestive of possible differential diagnosis including meningitis and retropharyngeal abscess. Subsequent radiological investigations led to a diagnosis of acute calcific tendonitis of the longus colli muscle. Typical clinical presentations and radiological findings of this rare condition are discussed.

## Introduction

Acute calcific tendonitis of the longus colli muscles, also known as retropharyngeal calcific tendonitis or prevertebral calcific tendonitis, is an uncommon benign and non-infective inflammatory process. It was first described by Hartley et al. in 1964 [1]. Since then, with more widely available imaging modalities, it has been described in several case reports over the last few decades [2-8]. In this report, we discuss a case of a young female who presented to the emergency department with acute neck pain associated with fever and odynophagia. These symptoms were concerning for diagnoses such as meningitis and retropharyngeal abscess. Subsequent imaging investigations lead to the diagnosis of acute calcific tendonitis of the longus colli muscle. 

## Case presentation

A 37-year-old female with no significant medical history presented to the emergency department with neck pain, odynophagia, and low-grade fever of one-day duration. Neck pain was localized over the upper paravertebral region and described as a squeezing pain associated with stiffness, made worst with neck movements. Review of systems did not reveal any symptoms of acute upper respiratory tract symptoms such as rhinorrhea or cough. She did not have a headache or photophobia.

Her chart revealed a low-grade fever (temperature of 37.7°C). Blood pressure, heart rate, and respiratory rates were within normal limits. Examination of her neck showed restriction of range of motion in all directions due to pain. Kernig’s and Brudzinski’s signs were negative. Examination of her oral cavity revealed a central uvula and did not reveal any oral ulcers, tonsillar exudates or infection. There were no palpable cervical lymph nodes or neck masses. There was no evidence of skin rash. Other joints were quiescent. Her neurological examination was otherwise unremarkable.

**Figure 1:**
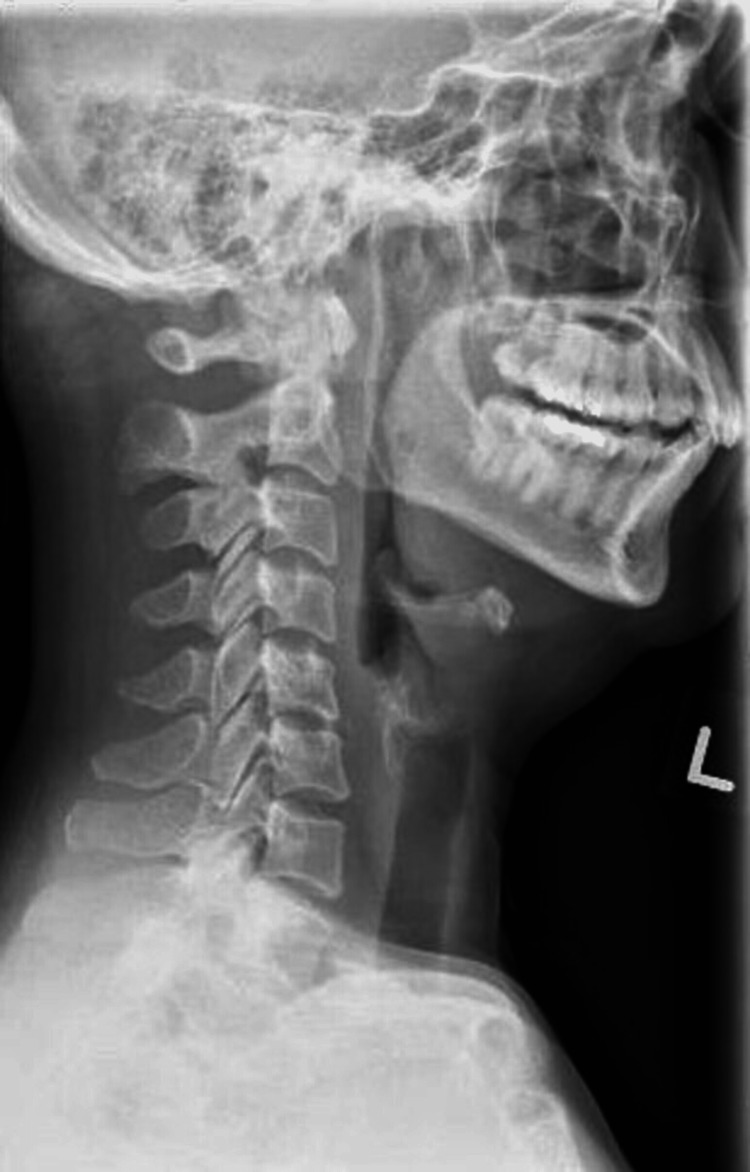
Lateral neck plain radiograph

The initial blood investigations revealed mild leukocytosis with neutrophilia (WBC total 11.15 x109/L, absolute neutrophil 8.01 x109/L) with a normal serum C-reactive protein (CRP) of 4.4 mg/L and procalcitonin of <0.06 µg/L). Her lateral neck radiograph (Figure 1) was unremarkable with no soft tissue swelling. She was empirically started on intravenous (IV) ceftriaxone due to an initial differential diagnosis of possible acute meningitis and retropharyngeal abscess.

**Figure 2:**
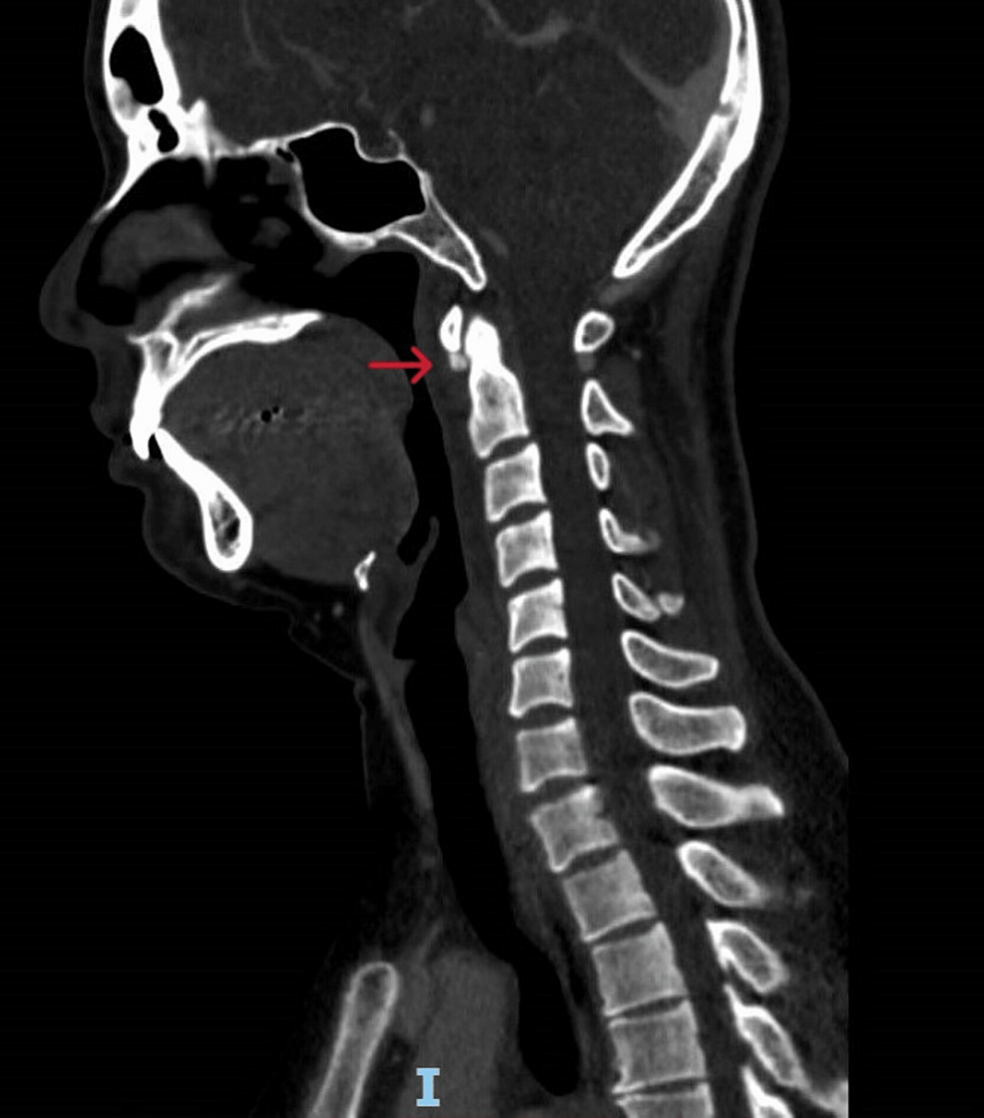
CT scan of the neck demonstrating an amorphic calcific density anterior to the C1 vertebra

**Figure 3:**
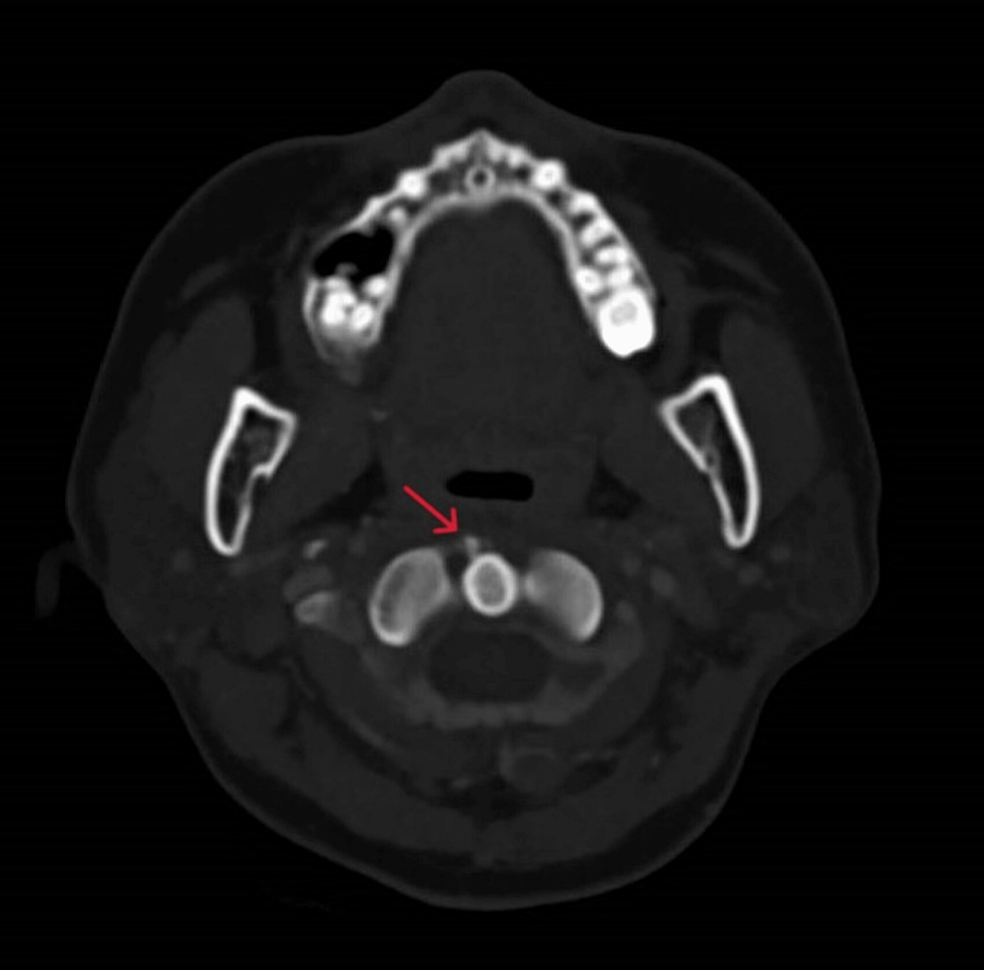
Calcific density on the axial cut anterior to the C1 vertebra

Given the atypical presentation, computer tomography (CT) scans of the neck and brain were obtained to look for local causes of infection or inflammation. The CT scan of the neck revealed a 5 mm calcification at the right inferior aspect of the first cervical vertebra (C1) anterior tubercle (Figures 2, 3). There was also a sliver of low-attenuation fluid in the retropharyngeal space, which was potentially reactive in nature. The adjacent longus colli muscles on both sides appeared slightly edematous. There were no fluid collections, and the other deep spaces of the neck did not reveal any infective foci. The CT scan of the brain was unremarkable.

A diagnosis of calcific tendinitis of the longus colli muscles was made. The patient was started on a course of non-steroidal anti-inflammatory drugs (NSAIDs) with etoricoxib for one week. Antibiotics were stopped and she was discharged from the hospital. The patient was reviewed in the outpatient setting a week later and had a complete resolution of her fever and neck pain.

## Discussion

The longus collis muscle spans between the C1 and T3 vertebrae and is a flexor of the neck and participates in rotation and tilt of the cervical vertebrae [9]. Zibis et al. described a characteristic triad of symptoms acute neck pain, neck stiffness, and odynophagia [2]. The latter is presumably caused by soft tissue swelling posterior to the pharynx. The exact incidence is not known but Boardman et al. found radiographic evidence of calcific tendonitis of the longus collis in 1.1 out of every 1000 CT studies [3].

The significance of this entity is that the clinical presentation can masquerade as other more sinister illnesses such as meningitis or retropharyngeal abscess [4]. Thus, it is important for clinicians to be aware of it to avoid unnecessary invasive investigations or treatment. In our patient, the symptoms of neck stiffness accompanied by low-grade fever raised concerns of meningitis. However, the lack of other symptoms such as headache made this diagnosis less likely. Our patient’s symptoms of odynophagia also raised concerns of a retropharyngeal abscess initially. However, the normal inflammatory markers and lateral neck radiograph made this diagnosis less likely as well.

The characteristic radiographic findings are prevertebral soft tissue swelling and a calcific density anterior to the C1-C2 vertebrae best seen on CT or MRI scans [5,10]. The lack of any rim-enhancing fluid collection can be helpful in differentiating a retropharyngeal abscess from the soft tissue swelling seen in acute calcific tendonitis [5,6].

The causative mechanism is the deposition of calcium hydroxyapatite crystals in the tendon causing reactive inflammation; however, the exact pathogenesis is still unknown [2,7,8]. Authors have hypothesized mechanisms such as degeneration because of tendon cell necrosis with calcium accumulation and erroneous differentiation of tendon-derived stem cells [11]. Another postulated mechanism described by Uhthoff et al. is the presence of fibro-cartilaginous metaplasia with a predilection for calcification during the formative phase. During a later resorptive phase, there is a breakdown of the cartilage due to an inflammatory response by phagocytes and mononuclear cells at the end of which there is calcium deposition and ossification [12].

Acute calcific tendonitis is a benign, self-limiting disease that in most cases can be managed with NSAIDs. In cases with more severe symptoms, corticosteroids or opioids may be considered as adjuncts. Most patients show improvement in symptoms after three days and symptom resolution is expected within one to two weeks [2,7,8]. No follow-up imaging is required if the diagnosis is certain, and patients improve over the expected clinical course.

## Conclusions

Acute calcific tendonitis is a benign, self-limiting, uncommon condition that clinicians may not be familiar with, given its rarity. Appropriate clinical suspicion and diagnosis with a CT neck can help to avoid invasive testing or treatment required in other illnesses with similar clinical presentations. Clinicians should also be mindful of the radiological findings and suspect alternative diagnoses if there is doubt or if a patient does not follow the expected clinical course.
